# Supplementary figures and images for: From musk to body odor: Decoding olfaction through genetic variation
Source: PLoS Genet. 2022 Feb 3;18(2):e1009564. doi: 10.1371/journal.pgen.1009564 (PMC8812863; doi:10.1371/journal.pgen.1009564)

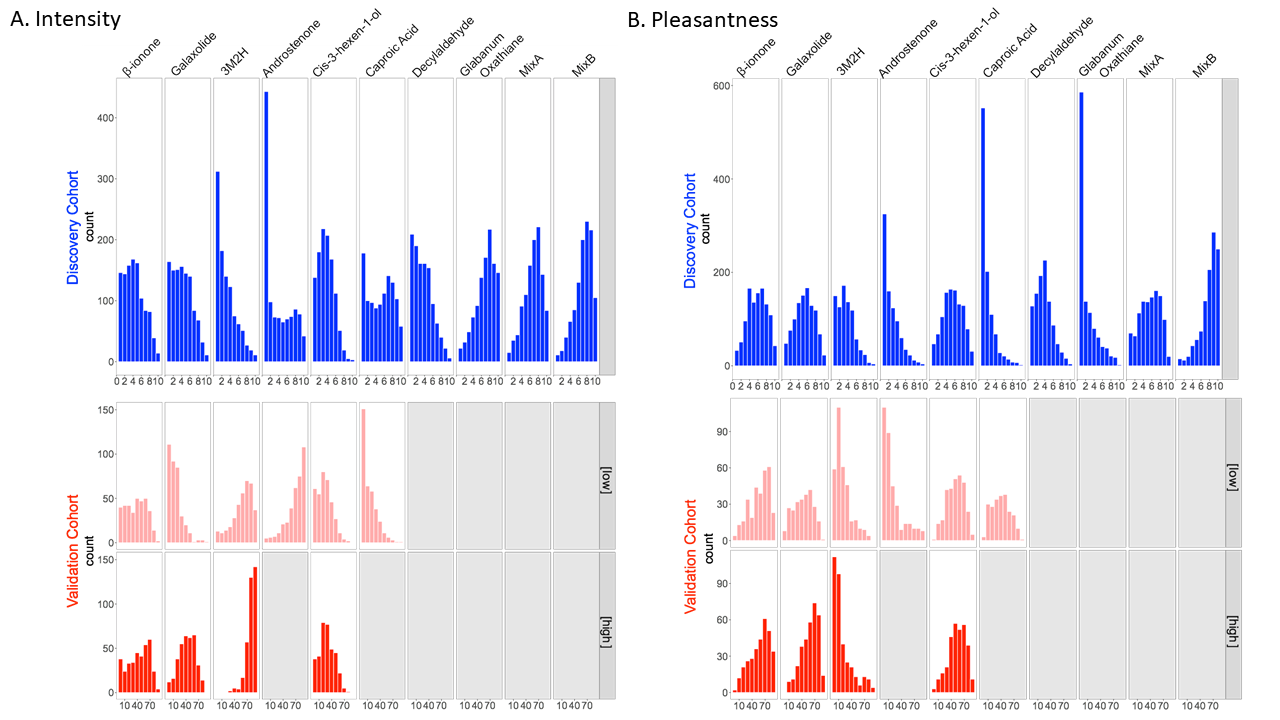

Supplement: S1 Fig — Distribution of ranked intensity (A) and pleasantness (B) ratings for odors in the discovery (blue) and replication (red) studies. A grey box indicates the phenotype was not tested. (TIF) [file pgen.1009564.s011.TIF]

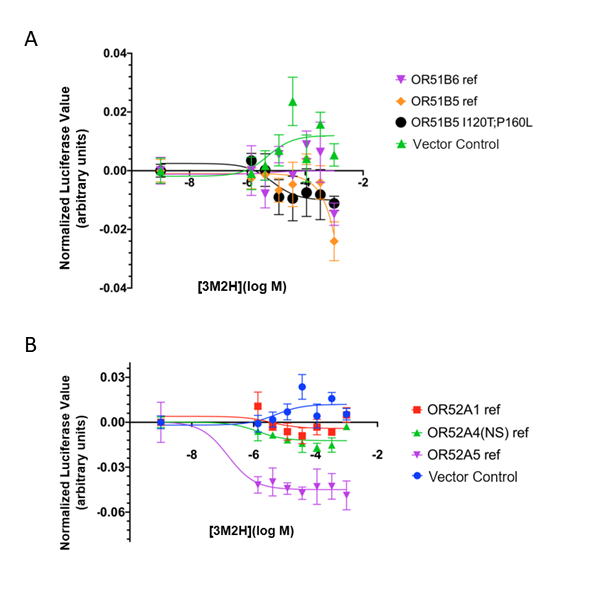

Supplement: S2 Fig — No receptors responded significantly above the vector control (Rho). Luciferase values were normalized by RL readings and then baselined to zero by subtracting the response of the no-odor control. (TIF) [file pgen.1009564.s012.tif]

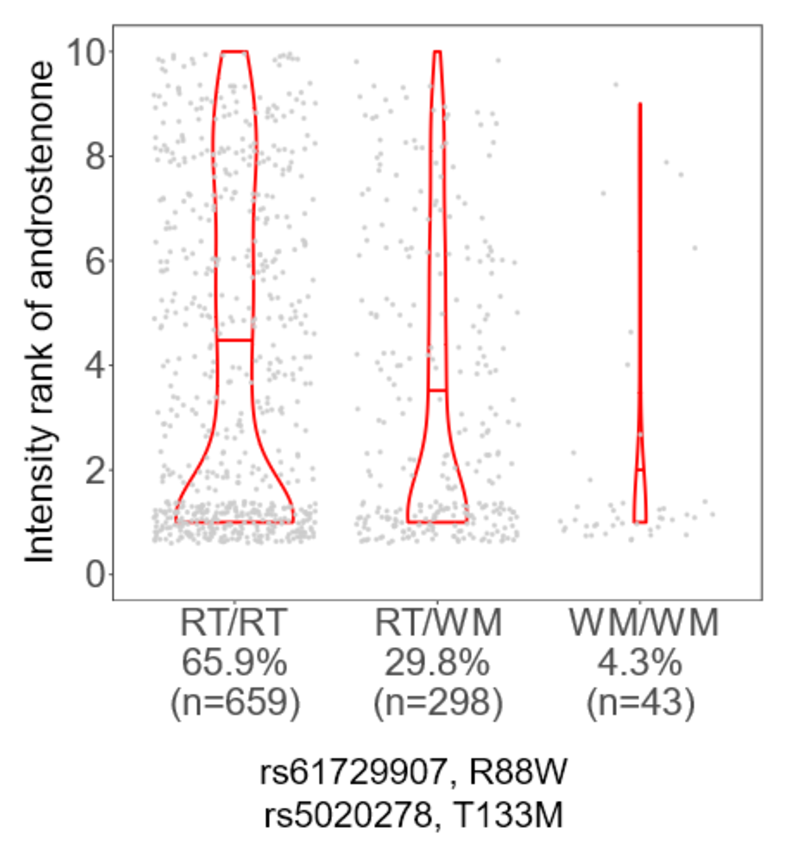

Supplement: S3 Fig — (TIF) [file pgen.1009564.s013.tif]

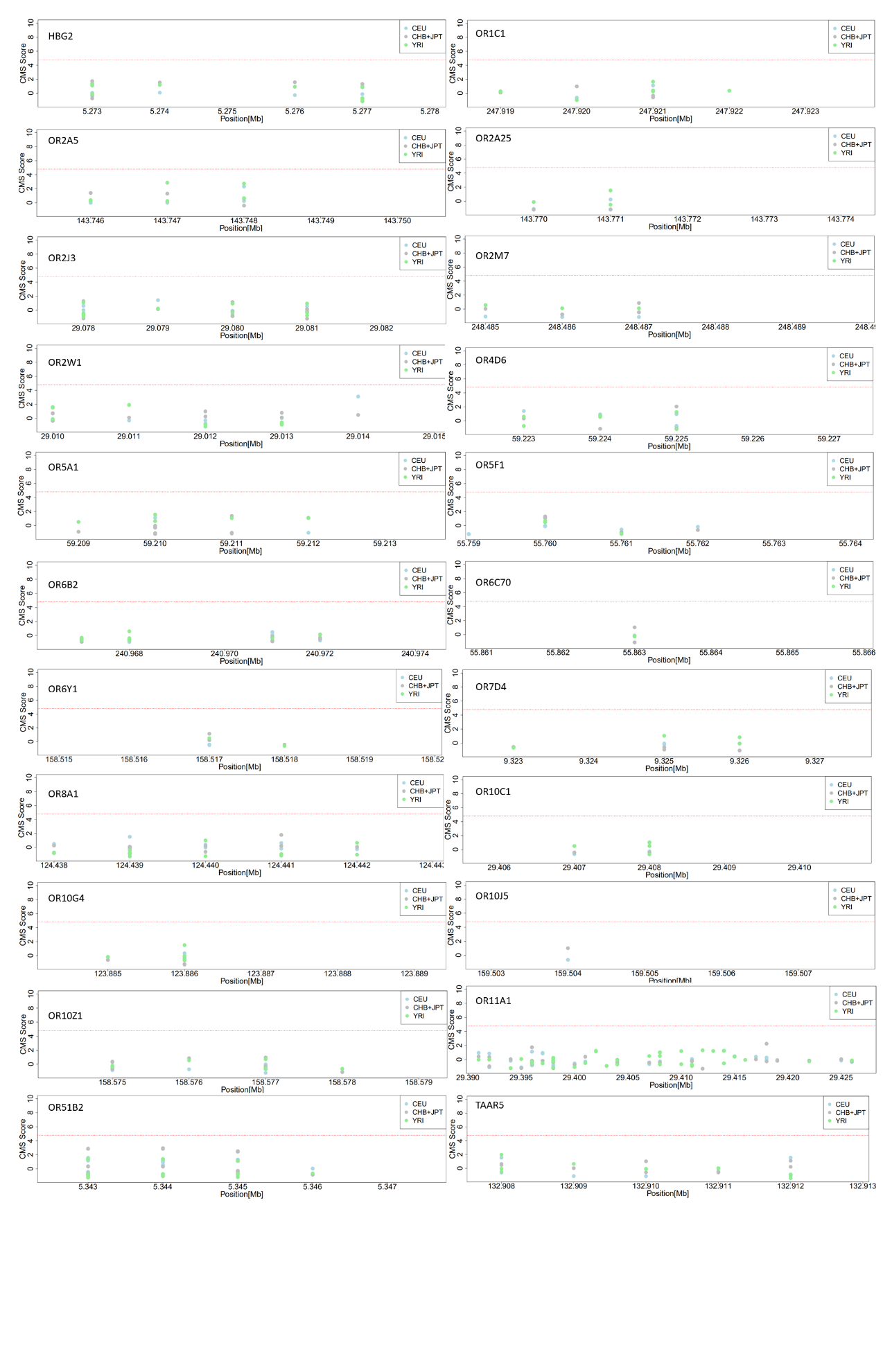

Supplement: S4 Fig — CMS scores are plotted against chromosome position in CEU, CHB+JPT, and YRI populations, shown in blue, gray, and green, respectively. The red dotted line represents the significance threshold (top 0.1% CMS score: 4.791). No enrichment for high CMS scores (top 0.1%) is found within the genes, indicating the examined SNPs are not subject to natural selection. (TIF) [file pgen.1009564.s014.tif]

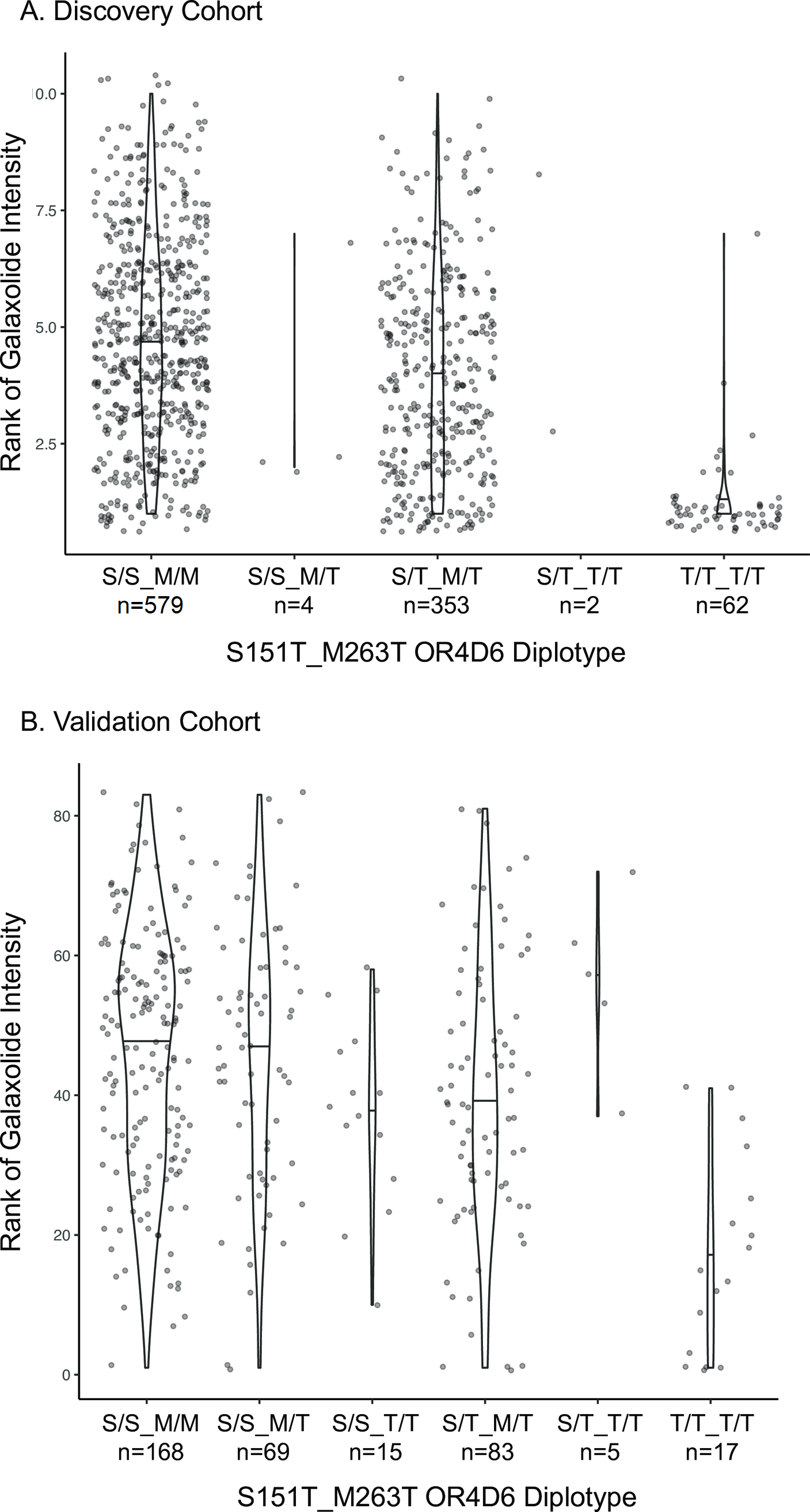

Supplement: S5 Fig — This shows the intensity of Galaxolide against the diplotypes for OR4D6 including the two significant SNPs M263T and S151T. In the discovery cohort, the higher LD between these two SNPs does not allow enough resolution to see which SNP is driving the association. In the replication cohort, where there is lower LD, it appears that the T/T genotype of S151T is driving the Galaxolide anosmia phenotype. (TIF) [file pgen.1009564.s015.tif]

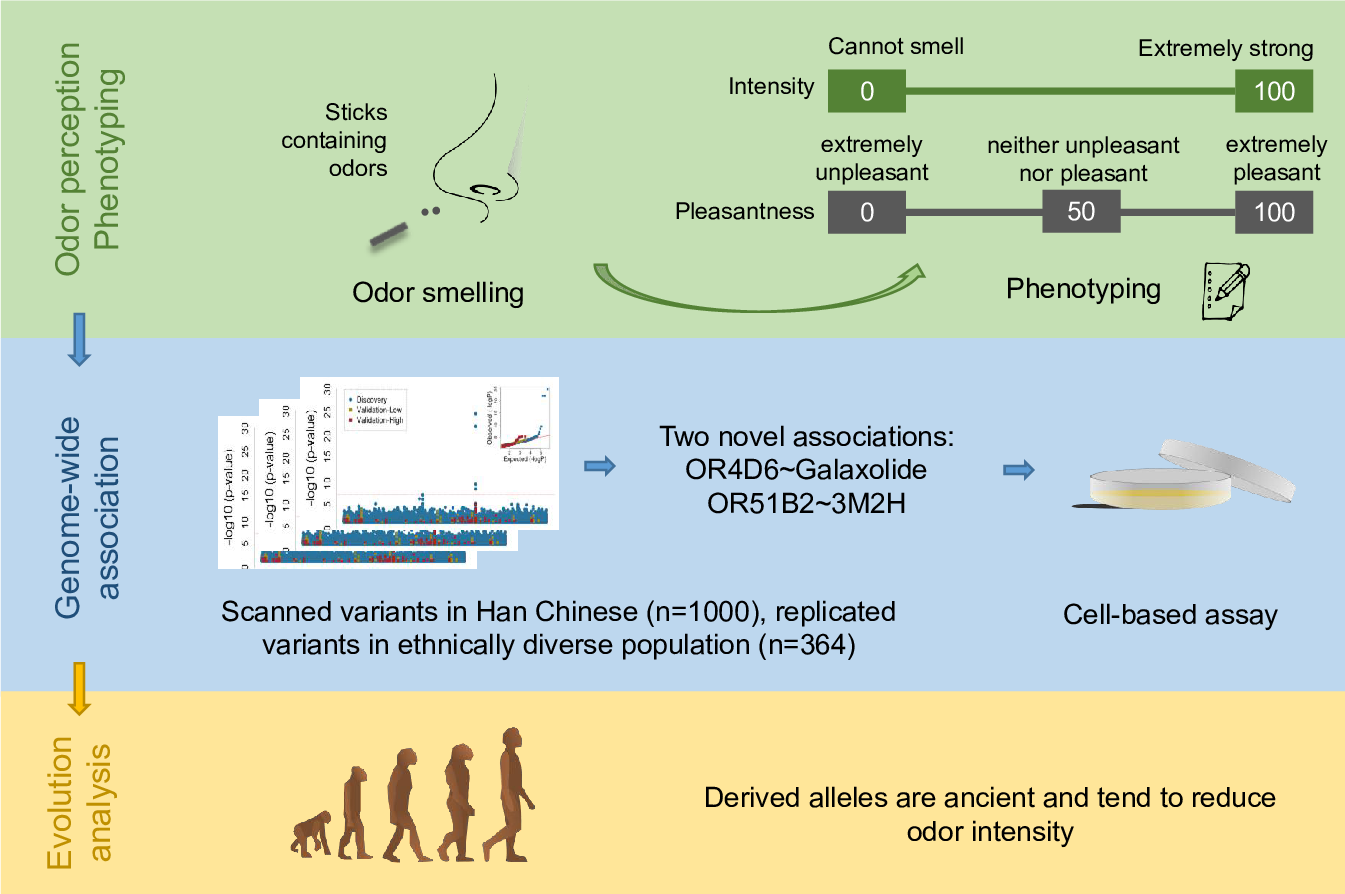

Supplement: S6 Fig — (TIF) [file pgen.1009564.s016.tif]

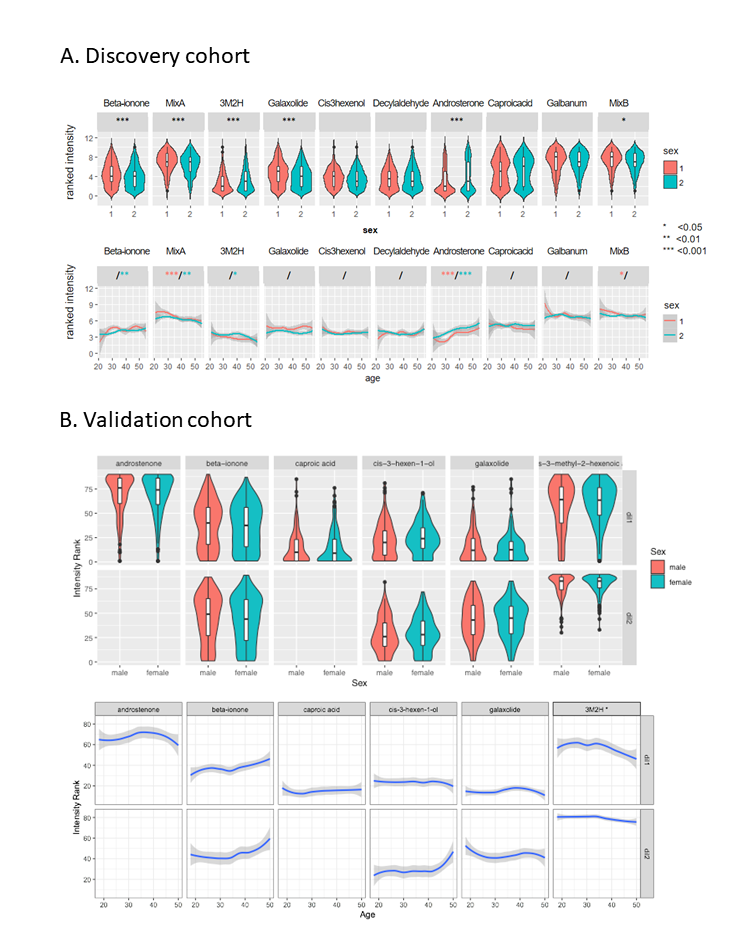

Supplement: S7 Fig — A) In the discovery cohort, several odors differ in intensity ranking between sexes and/or across age (Male = 1, Female = 2). B) In the validation cohort, there are no significant differences in odor intensity across sex. 3M2H intensity (at both concentration of the odor) significantly decreases as age increases after Bonferroni correction (p <0.002 for low concentration (dil1); p < 0.002 for high concentration (dil2)). For both cohorts, sex effects were tested using a t-test, and age effects were tested with a linear model. We used sex as a covariate in the linear model testing for age effects in the discovery cohort, but not the validation cohort, due to significant effects on sex in odor perception in the discovery cohort. (TIF) [file pgen.1009564.s017.tif]
