# Supplementary material for: From musk to body odor: Decoding olfaction through genetic variation
Source: PLoS Genet. 2022 Feb 3;18(2):e1009564. doi: 10.1371/journal.pgen.1009564 (PMC8812863; doi:10.1371/journal.pgen.1009564)
Supplement: Attachment — Submitted filename: review report.docx [file pgen.1009564.s018.docx]

The authors conduct a GWAS using ten odors in 1003 Han Chinses and found novel associations for OR4D6 with the musk odor Galaxolide, and OR51B2 with 3M2H. Furthermore, they validate their results in a multi-ethnic independent population and different methodology, which demonstrates these associations are stable across populations and robust to differences in methods, including odor concentration and delivery method. Although the manuscript provides an interesting topic of public concern, there are several major concerns.

1. As shown in supplemental figure 1, some of the phenotypes are not normally distributed. The authors should provide more details on how they did normalization in association analysis. rank-based inverse normal transformation? log transformation?

2. The within-subject reliability of rating is acceptable in discovery cohort (test-retest r=0.75). However, the consistency in replication cohort is relatively low (test-retest r=0.69). I’m concerned that these subjective rating systems may bring bias results. Please mention this in the discussion/limitations.

3. The article has been submitted to a genetics journal but I suspect the current explanation of the methods may rely too much on familiarity with studies of Neuroscience. No doubt the authors can fix this. A graphic abstract may help the reader gain an overview of this study easily.

4. Is there a typo in ‘n both cohorts, all the genetic variants that were significantly associated with pleasantness were also significantly associated with intensity perception.’?
