# Supplementary material for: From musk to body odor: Decoding olfaction through genetic variation
Source: PLoS Genet. 2022 Feb 3;18(2):e1009564. doi: 10.1371/journal.pgen.1009564 (PMC8812863; doi:10.1371/journal.pgen.1009564)
Supplement: Attachment — Submitted filename: response_letter.docx [file pgen.1009564.s019.docx]

Reviewer #1: Review of Li et al.
The authors describe an extensive study of specific anosmias for a range of compounds initially in Han Chinese, finding their genetic basis through GWA and cell-based assays. Their specific discoveries are around the odorant receptor genes harbouring polymorphisms explaining differences in the intensity rating of musk compounds. This class of compounds is important as they are thought to potentially underpin certain behaviours in humans from kin to mate recognition. The authors also reconfirm a number of previously described associations between odorants and odorant receptor genes and also demonstrate that in the main loss of ability to smell many of these compounds is a derived state. These findings lead them to the conclusion that humans have been gradually increasing the number of segregating polymorphism for alleles that encode reduced ability to detect many odorants.
The authors have conducted a well thought out set of phenotyping regimes to compare with SNP data and have also replicated their finding in another population. And cell-based assays were successful in confirming one of the two new gene/odorant associations. The findings are significant to the field adding to our knowledge of the receptors underpinning musk perception. I have only a few suggestions on how the manuscript could be improved.

1. It would be good to add information on the origin and purity of the odorants used to the methods section.

*Response: Thank you for the suggestion. We have added a Supplementary Table S7 that includes information with the CAS and catalogue numbers for monomolecular odorants used in the study. All monomolecular odors used had >96% purity, with the exception of Galaxolide which had >85% purity in the replication, and was ordered in a 50% mixture solution in the discovery (48-52% purity). We cannot provide further information about the two odor mixtures, as they are proprietary to the provided suppliers. It is noted in line 610 on page 28 of the manuscript.*

2. Also it was not clear to me on the approach used in reusing data on the statistical treatment. Was an FDR approach or similar correction used for the GWA statistics?

*Response: The genome-wide significance threshold is 5x10^-8^ for GWAS as a convention, even when multiple phenotypes are tested. Some examples are listed below. It is now described in line 714 on page 34 of the manuscript.*

- *PLoS Genet, 2017:* *https://www.ncbi.nlm.nih.gov/pubmed/?term=28787443, 15 hematological phenotypes.*
- *Nat Commun, 2019:* *https://www.ncbi.nlm.nih.gov/pubmed/?term=30664655, 6 pigmentation phenotypes.*

*The most stringent threshold is P<2.5x10^-9^(5x10^-8^/20) by further applying a Bonferroni correction based on the number of studied phenotypes. Even under this most stringent threshold, our main results are actually still significant (Galaxolide/rs1453541 and rs1453542 (p<**2.1x10^-25^, p<**2.6x10^-25^) and 3M2H/rs3898917 and rs10837814 (p<**1.2x10^-11^, p<6.57x10^-10^).*

3. The authors claim that this is the first undertaking of this type of study in Asian populations. McRae et al. (ref 34) did conduct a replication experiment within their 2013 study in SE Asians.

*Response: Thank you for pointing out this oversight. We have rephrased the relevant parts of the manuscript to focus on the size and homogeneity of the discovery cohort population, which to our knowledge, has not been seen before in this type of research. This is reflected in the following changes:*

- *Line 92, page 5 (Introduction): Rephrased from “unique” to “understudied”.*
- *Line 445, page 21: Removed “for the first time” from the sentence.*
- *Lines 485-490, pages 23: Changed focus from novelty of East Asian population to general lack of information about non-majority European populations.*

4. The authors might wish to consider also looking at the associations among the different polymorphisms across their study participants. How independent were they?

*Response:* *Thank you for the comment. We looked at the linkage disequilibrium (LD) between the most significant SNPs associated with galaxolide, 3M2H, and beta-ionone in both cohorts. While the 3M2H-associated rs10837814 is not in LD with the other two SNPs, the Beta-ionone-associated rs6591536 and Galaxolide-associated rs1453541 are in LD (R^2^=0.72), as they are only 14kb apart (Table I). The SNP rs6591536 was functionally validated to be the causal variant affecting the perception of Beta-ionone. (Jaeger et al.2013*,*ref 9 in the manuscript)* *However, it is unlikely to be the causal variant affecting the perception of Galaxolide for the following reasons: 1) fine-mapping using PAINTOR found the 99% credible set only contain SNPs rs1453541 and rs1453542, with the posterior probability of 58% and 42%, respectively. The probability of rs6591536 being the causal variant is close to zero. 2) We have performed cell-based assay on a number of SNPs, including rs6591536, but found no significant response (Supplementary Table 5). 3) If rs6591536 was indeed the causal SNP for the perception of Galaxolide, one would expect the two phenotypes (perception of Galaxolide and Beta-ionone) should be correlated to some extent, but this is not the case (ρ=-0.01, Pearson’s correlation). We discuss these points in lines 391-402 on page 19 of the manuscript.*

*Table I. LD information between the most significant SNPs associated with galaxolide, 3M2H, and beta-ionone.*

| *Odor1* | *SNP1* | *Chr:BP*  *(hg19)* | *Odor2* | *SNP2* | *Chr:BP*  *(hg19)* | *R^2^*  *(discovery)* | *R^2^*  *(replication)* |
| --- | --- | --- | --- | --- | --- | --- | --- |
| *3M2H* | *rs10837814* | *11:* *5345128* | *Beta-ionone* | *rs6591536* | *11: 59211188* | *<0.01* | *0.04* |
| *3M2H* | *rs10837814* | *11: 5345128* | *Galaxolide* | *rs1453541* | *11: 59225221* | *<0.01* | *0.01* |
| *Beta-ionone* | *rs6591536* | *11: 59211188* | *Galaxolide* | *rs1453541* | *11: 59225221* | *0.72* | *0.48* |

Minor points

5. The legend of Fig 1 has a sentence that needs attention. The fourth sentence … “ but not did examine” doesn’t make sense.

*Response: The Figure 1 referred to in this comment is now Figure 2. We have clarified the wording in this figure legend in lines 1056-1058 on page 45 of the manuscript.*

6. The last sentence of paragraph 1 on page 3 needs an “I” added to “In”

*Response: We have corrected it in line 185 on page 9 of the manuscript.*

7. On page 11 in the Odor concentration and preparation section “difference” needs to be changed to “different”.

*Response: We have corrected it in line 614 on page 29 of the manuscript.*

Reviewer #2: Li, Kamarck, Peng and colleagues examine the association between genetic variation in olfactory receptors and perception of 10 odors. They report improvements on previous methods (larger population size, a non-Western and homogenous study population, and inclusion of a validation study). Though the latter is a standard in the field, it is rarely successfully utilized for olfactory genetic studies. The authors discover novel associations for two interesting odors: a musk, which is a group of odors for which the coding of perceptual information has been difficult to determine, and a major contributor to body odor, which may have importance for human chemical communication. The results are of general interest for the study of large multi-gene families in which similar information, i.e. the “musk percept,” may be encoded by different receptors. Their analysis is statistically sound and the figures and tables are clear. Further clarification on a few questions is requested below:

1. Figure 1 does not indicate whether the association is with intensity or pleasantness, which might be interesting information to illustrate.

*Response: Thank you for pointing out this issue. The Figure 1 referred to in this comment is now Figure 2. This figure is indicating only associations with intensity phenotypes. We have added this information in the figure title and legend in lines 1047-1048 on page 45 of the manuscript.*

2. Given that the authors have phased haplotype information for OR4D6, it might be useful to add a figure (or a supplementary figure) on what galaxolide perception looks like for the different SNP combinations in their study populations. It appears the LD between M263T and S151T is different in the discovery and validation cohorts.

*Response:* *Thank you for the suggestion. We have added the following as Supplementary Figure 5. This shows the intensity of Galaxolide against the diplotypes for M263T and S151T in OR4D6. In the discovery cohort, the higher LD between these two SNPs does not allow enough resolution to see which SNPs is driving the association. In the replication cohort, where there is lower LD, it appears that the T/T genotype of S151T is driving the Galaxolide anosmia phenomenon. It is noted in line 390 on page 19 of the manuscript.*

3. SI Tables S5 and S6 would benefit from including population frequency for these haplotypes.

*Response: We have added population frequency for these haplotypes in Supplementary Tables 5 and 6.*

4. The manuscript lacks a mechanistic explanation for the association with OR51B2, i.e. they fail to tie functional differences in this OR to differences in 3M2H perception. The associated variant, L134F, is found at a high frequency in the population in combination with other amino acid changes. Given the effect reported, one would hypothesize the F variant will increase receptor function. These variants should be tested or an explanation provided for why they weren’t examined.

*Response: We tested a variant haplotype containing L134F against 3M2H but did not observe a response. This result is described on page 12, lines 251-253 of the manuscript, and the graph for Figure 4e has been changed to reflect the results of this new experiment (page 47, line 1092). In addition, we have added haplotype frequency information about the newly tested haplotype to Supplementary Table 6. Although we were surprised by this result, this assay has previously failed to provide direct support for genetic associations (Trimmer et al., 2019, ref 10 in manuscript). We have added explanation to the discussion to address this point (pages 20-21, lines 426-435).*

5. There is a large difference between published data and what is reported here in the percent variation explained in beta ionone perception – 96% published for sensitivity and approximately 22% and 32% here for intensity. Could this have implications for coding of beta ionone information, i.e. more receptors are recruited for intensity perception?

*Response: This is a great point, but it is hard to draw conclusions where the different concentrations and phenotyping methods (threshold in* *Jaeger et al.2013, ref 9 in the manuscript vs. suprathreshold intensity in this study) were run across two different studies. For example, the Jaeger et al. 2013 study tested 163 Caucasian participants at three concentrations (0.22, 2.2 and 22.0 ppm), which may impact the set of SNPs that influence behavior and is difficult to dissociate from any concentration effect. Here, we focused on the association between SNPs and behaviors under only one concentration each time, by ranking order across 10 odors. In summary, while we do highly appreciate the interesting point brought up here, the different design means it is out of the scope of this study to draw any reasonable conclusions about the number of receptors playing a role at different concentrations.*

6. In the introduction, the authors mention that one improvement to their methodology is using a non-Western and homogenous, i.e. Han Chinese population, for their discovery cohort. But this point is not mentioned later in the manuscript. The authors may want to consider adding a bit more on the benefit of studying a unique (non-Caucasian) cohort and whether its genetic similarity is beneficial to their discoveries.

*Response: Thank you for reminding us of revisiting the point made in the introduction (use of a homogenous population). It is not easy to clearly understand whether the novel findings were due to the choice of population, or the larger sample size, or a combination of both. Since this study was not designed to figure this problem out, so we decide to weaken the point a bit in the introduction.* *In this study,* *the novel signals do not have much population differences in MAF or effect size (Table 1, Figure 3-4), suggesting that the large sample size rather than its genetic similarity might be the more important reason behind the findings.*

*To further test this point, we randomly sampled 364 (same as the sample size of the validation cohort) individuals from the discovery cohort, and re-calculated the P values of top associations. This analysis was repeated 1000 times to obtain a distribution of P values (Figure Ⅰ), so that we could statistically compare this results of down-sampled discovery cohort with that of the validation cohort.* *We can see that, in the case of the same sample size, the more homogenous discovery cohort showed more significant association for galaxolide (P<0.05), but less significant association for 3M2H (P<0.05), when compared with the validation cohort. This indicates that large sample size is probably the main reason that we could find a very significant signal for 3M2H in the full discovery cohort, but it might not be the main reason in the case of Galaxolide, where genetic similarity may provide some benefits.*

*This is reflected in the following changes:*

- *Line 92, page 5 (introduction): Remove the “homogenous” from the sentence.*
- *Lines 537-539, page 25: Add some related discussions.*

*Figure Ⅰ* *The distribution of P values of the novel SNPs in the discovery cohort with a small sample size (N=364).*

An interesting analysis (and associated figures) for quantifying genetic distance is discussed only in the methods section and illustrated in supplemental, so interested readers may miss these points.

*Response: Thanks for the suggestion. We have moved the genetic distance analysis back to the first paragraph of results section and main figures to help highlight this point. It is now described in lines 153-164 on pages 7-8 of the manuscript.*

Along these lines, the distribution of 3M2H perceived intensity was very different among discovery and validation study populations. The authors note in the discussion that allele frequency for OR51B2 is similar among populations, meaning genetic differences cannot provide an explanation. Is this an example of diversity in study population being beneficial?

*Response: The current version of the discussion does not include mention of a difference in allele frequency for OR51B2. Table 1 shows that allele frequency for OR51B2 is not similar between the population (EAS=0.68, EUR=0.39). It is possible that the difference in allele frequency contributes to the difference in distributions of 3M2H intensity. It is also worth noting that the two studies used different methods for delivering the odors as well as different concentrations of odor, both of which likely contributed to the difference in phenotype distribution. Due to the high degree of replicability in both our homogeneous discovery cohort and our heterogeneous validation cohort, we suggest that a large number of people in a sample could compensate diversity in the population at some extent. This is exemplified by the recent paper by Gisladottir et al., 2019, ref 11 in the manuscript. They were able to detect a rare genetic variation in TAAR5 that related to fishy perception due to their large, but homogeneous Icelandic population. In their case, this variant happened to be the most prevalent in their population of choice, which may have increased their ability to detect a signal. We added this discussion in lines 438-441 on page 21 of the manuscript.*

7. There is no discussion of the effect of age or sex on odor perception – were these examined with no effect or not considered in the study?

*Response: Thank you for bringing up this question. We previously mentioned that the discovery cohort included age and sex as covariates in the GWAS model, as described in lines 712-713 on page 34 of the manuscript.* *To provide further information on age and sex we added the following figure as Supplementary Figure 7. For several odors in the discovery cohort, there are significant differences between sexes (beta-ionone, 3M2H, Galaxolide, etc.). Similarly, the intensity of several odors varies with age (Beta-ionone, 3M2H, Androstenone, etc.).*

*The validation cohort did not use age and sex as covariates as these factors were largely non-significant. There were no significant effects for sex, and only 3M2H was significant for age such that intensity perception of 3M2H decreased with age (see graph below which is now part of supplemental Figure 7). Here, we did not use sex as a covariate for the age analysis as it was not a significant factor.*

*Besides, we found that in the validation cohort, using age and sex as covariates did not change the outcome of any of our significant findings (see Table Ⅱ p values).*

*Table Ⅱ Comparison of main results before and after adding age and sex as covariates.*

| *CHR* | *BP* | *SNP* | *Task* | *Odor* | *OR_name* | *P_value_manuscript* | *P_value_withAgeSexCOV* |
| --- | --- | --- | --- | --- | --- | --- | --- |
| *11* | *59225221* | *rs1453541* | *Intensity* | *Galaxolide* | *OR4D6* | *9.079e-06* | *1.197e-05* |
| *11* | *59224885* | *rs1453542* | *Intensity* | *Galaxolide* | *OR4D6* | *1.020e-05* | *1.599e-05* |
| *11* | *5345128* | *rs10837814* | *Intensity* | *3M2H* | *OR51B2* | *9.599e-08* | *1.399e-07* |
| *11* | *59211188* | *rs6591536* | *Intensity* | *Beta-ionone* | *OR5A1* | *7.821e-39* | *1.087e-37* |
| *19* | *9325278* | *NA* | *Intensity* | *Androstenone* | *OR7D4* | *3.533e-04* | *3.780e-04* |
| *17* | *3119611* | *NA* | *Intensity* | *Caproic&acid* | *OR1A1* | *6.943e-03* | *8.221e-03* |

8. A brief sentence on some of the methods used would be useful. For example what constitutes the “credible set” or what a CMS score is (the acronym is undefined for its first use in the manuscript).

*Response: It is now described in line 324 on page 15, lines 743-746 on page 35, and lines 807-809 on page 38 of the manuscript.*

9. Why were different quality control cutoffs used for the discovery and validation cohort, i.e. a MAF of 1% for discovery and 5% for validation?

*Response: The different cutoffs are due to the consideration of different sample sizes. We adjusted the cutoffs appropriately in the validation cohort as the sample size of the validation cohort is smaller than the discovery cohort. It is now described in lines 676-678 on page 32 in the manuscript.*

Reviewer #3: The authors conduct a GWAS using ten odors in 1003 Han Chinses and found novel associations for OR4D6 with the musk odor Galaxolide, and OR51B2 with 3M2H. Furthermore, they validate their results in a multi-ethnic independent population and different methodology, which demonstrates these associations are stable across populations and robust to differences in methods, including odor concentration and delivery method. Although the manuscript provides an interesting topic of public concern, there are several major concerns.

1. As shown in supplemental figure 1, some of the phenotypes are not normally distributed. The authors should provide more details on how they did normalization in association analysis. rank-based inverse normal transformation? log transformation?

*Response: The data was normalized by rank (described in lines 590-596 on page 28 of the manuscript), but not further transformed. This is in line with previous publication in this field (Keller et al., 2007, ref 6 in the manuscript). To test whether our results would be affected by further transformation, we performed rank-based inverse normal transformation on both sets of data and re-ran the analyses. The major findings remained the same after transformation (Figure Ⅱ, Table III).*

*Figure Ⅱ No new signals were found for GWAS using the transformed data.*

*Table Ⅲ Comparison of main results before and after performing the further transformation.*

A) *Discovery cohort*

| *CHR* | *BP* | *SNP* | *Task* | *Odor* | *OR*  *name* | *P_manuscript* | *P_transformed* |
| --- | --- | --- | --- | --- | --- | --- | --- |
| *11* | *59225221* | *rs1453541* | *intensity* | *galaxolide* | *OR4D6* | *2.07x10^-25^* | *5.78x10^-34^* |
| *11* | *59224885* | *rs1453542* | *intensity* | *galaxolide* | *OR4D6* | *2.59x10^-25^* | *4.86x10^-35^* |
| *11* | *5345128* | *rs10837814* | *intensity* | *Trans-3-methyl-2-hexenoic&acid* | *OR51B2* | *6.57x10^-10^* | *6.16x10^-12^* |
| *11* | *59211188* | *rs6591536* | *intensity* | *Beta-ionone* | *OR5A1* | *1.84x10^-41^* | *2.90x10^-40^* |

*B) Validation cohort*

| *CHR* | *BP* | *SNP* | *Task* | *Odor* | *OR*  *name* | *P_manuscript* | *P_transformed* |
| --- | --- | --- | --- | --- | --- | --- | --- |
| *11* | *59225221* | *rs1453541* | *intensity* | *galaxolide* | *OR4D6* | *9.08x10^-6^* | *4.95x10^-4^* |
| *11* | *59224885* | *rs1453542* | *intensity* | *galaxolide* | *OR4D6* | *1.02x10^-5^* | *9.38x10^-5^* |
| *11* | *5345128* | *rs10837814* | *intensity* | *Trans-3-methyl-2-hexenoic&acid* | *OR51B2* | *9.60x10^-8^* | *3.69x10^-7^* |
| *11* | *59211188* | *rs6591536* | *intensity* | *Beta-ionone* | *OR5A1* | *7.82x10^-39^* | *4.33x10^-30^* |

2. The within-subject reliability of rating is acceptable in discovery cohort (test-retest r=0.75). However, the consistency in replication cohort is relatively low (test-retest r=0.69). I’m concerned that these subjective rating systems may bring bias results. Please mention this in the discussion/limitations.

*Response: Thank you for pointing out this issue. Test retest is often used to measure within-subject reliability of ratings. A low test-retest reliability can limit the predictive performance of a model, such that it serves as the upper limit for model performance. Simply put, a SNP cannot do a better job of predicting a subject’s rating than the subjects themselves. We have included in lines 472-477 of pages 22-23 of the discussion the limitations of SNPs predicted variance due to these test-retest scores. Additionally, we suggest that in the future, we could employ more training in order to improve subject’s reliability and increase our explanatory power.*

*The replication cohort had a test retest correlation of r=0.69, which is lower than in the discovery study, but might be considered high within the larger field of olfactory psychophysics. For example, in a paper by Keller et al., 2017 (see below), a highly predictive model was built from data with test-retest reliability scores ranging from ~0.1-~0.6.*

- *Keller et al. (2017). Predicting human olfactory perception from chemical features of odor molecules. Science, 355:820-826. 10.1126/science.aal2014.*

*To determine if the within-subject reliability was significantly different between these populations, we ran a pairwise t-test on the test-retest correlations by odor and task (intensity/pleasantness). We found that across the 12 matching phenotypes (see Figure III below), these two cohorts were not significantly different (p=0.12).*

*Figure III Comparison of test-retest correlation between the two cohorts.*

3. The article has been submitted to a genetics journal but I suspect the current explanation of the methods may rely too much on familiarity with studies of Neuroscience. No doubt the authors can fix this. A graphic abstract may help the reader gain an overview of this study easily.

*Response: We made a graphic abstract to help the reader gain an overview of this study easily (see the following figure), and added it as Supplementary Figure 6.*

4. Is there a typo in ‘n both cohorts, all the genetic variants that were significantly associated with pleasantness were also significantly associated with intensity perception.’?

*Response: We have corrected it in line 185 on page 9 of the manuscript.*
