# Supplementary material for: From musk to body odor: Decoding olfaction through genetic variation
Source: PLoS Genet. 2022 Feb 3;18(2):e1009564. doi: 10.1371/journal.pgen.1009564 (PMC8812863; doi:10.1371/journal.pgen.1009564)
Supplement: Attachment — Submitted filename: Response letter.docx [file pgen.1009564.s020.docx]

Reviewer's Responses to Questions

**Comments to the Authors:
Please note here if the review is uploaded as an attachment.**

Reviewer #1: I'm satisfied that the authors are made the necessary changes to the manuscript.

Reviewer #2: The authors responded thoroughly to all questions and suggestions for clarity! A few points:

1 – Thank you for providing the additional diplotype graphs! To clarify my earlier suggestion, I was interested in the effect of each haplotype on perceived intensity. Subjects are mainly reference (SM) or TT, although, as noted in your reply, this is more interesting in the validation cohort.

*Response: Thank you for clarifying this point. In the validation cohort, there are 23 unique haplotypes for OR4D6, most of which are rare. Below we have graphed Galaxolide intensity against the haplotypes of OR4D6 that exist in greater than 5% frequency in our population. In the validation cohort these are: reference (9.1%), D96G (53.0%), D96G; F102S; M263T (5.2%), and M59V; D96G; S151T; M263T (12.5%). In this graph, each subject can be represented up to two times for the two inherited haplotypes. In the validation cohort, the high frequency haplotypes result in 581 data points out of a potential the original 728 (364 subjects x 2).* *In the discovery cohort, there are only 5 variants in OR4D6, 4 of which are present in the haplotypes mentioned above* *(D96G, M263T, M59V, S151T). So we performed the haplotype analysis based on these 4 variants (D96G, M263T, M59V, S151T) and found 5 unique haplotypes, 4 of which are greater than 5% frequency. These are reference (6.63%), D96G (69.19%), D96G;S151T;M263T (8.52%), and M59V;D96G;S151T;M263T (15.35%).*

*Although we observe a small decrease in intensity for the haplotypes containing M263T and S151T, the Galaxolide anosmia phenotype is best observed with the diplotype (supplementary figure 5) where we can observe the effect in individuals possessing two copies of S151T.* *So, we hope the reviewer could agree with us to keep the diplotype graph (not the haplotype graph) as Supplementary Figure 5.*

2 – Thank you for the additional work on the OR51B2/4 cluster. The legend for panel e indicates you tested OR51B2 L134F, but in the table describing tested variants you have the full haplotype (C120R; L134F; C209S). Did you test one amino acid change or all three? Also, please align with how the haplotype for OR51B4 is designated – changes in amino acid only vs. amino acid number.

*Response: Thank you for pointing out this discrepancy. In cells, we tested the full haplotype (C120R; L134F; C209S). We have fixed the legend for Figure 4e to fix this error and to format the other haplotypes by amino acid with number. We have also clarified this in the figure legend for 4e (****line 1096****).*

3 – Line 45 needs a “1” in “OR5A1.”

*Response: Thank you for pointing it out. We have made this correction (****line 46****).*

4 – In the legend for Figure 3, is 0.02 the true value for the genomic lambda of galaxolide in the discovery cohort?

*Response: Thank you for finding this error. The genomic lambda value for Galaxolide in the discovery cohort is actually 1.02. We have made this correction in the manuscript (****line 1077****).*

Reviewer #4: The manuscript by Li and colleagues reports a GWAS study where individual differences in the perception of a set of odors was found to be associated with genotypic variation. This reviewer was asked to evaluate the suitability of the revised manuscript for publication following a previous round of revisions that included three other reviewers. The following evaluation thus takes into account the author replies, as well as the revised manuscript.

All three previous reviewers posed insightful and in-depth comments on the present work, focusing mostly on methodological aspects related to genetic measurement and statistical assessment. Importantly, the authors provided thoughtful and elaborate replies to these comments, and the revisions were appropriate. Although I have identified some additional issues (listed below), they may be considered minor and relate mostly to the interpretation and presentation of the findings. I do not see any remaining major flaw in the revised version of the manuscript, and it is very well written overall.

1. Two of the main limitations of the present work are that (a) some odors for which there appears to have been strong a priori hypotheses (e.g. two aldehydes and MixA and MixB), did not give clear-cut results, which is hard to explain. This should be mentioned in the abstract. On the other hand, some novel results were discovered and validated across samples, they constitute significant contributions to the field and outweigh the limitations. Moreover (b) that odor perception assessments are somewhat different across samples. This makes discrepant results across samples difficult to interpret. These issues are not, in my view, obstacles to publication, but they should be highlighted and discussed to provide an appropriate context and transparency.

*Response: a) Thank you for the suggestion. Prior to our study there was shown to be perceptual variability in aldehydes, but we did not know if this would be due to genetic differences. Likewise, for the mixtures, we were aware of cultural variability in the perception of the mixtures, but did not have a strong hypothesis that this would necessarily be due to genetic factors. We mention in our discussion (lines 524-525) that these differences could be due to factors other than genetics, such as cultural differences. Previous literature has also had less success in finding genetic associations with mixtures than with monomolecular odorants, potentially due to compensation by other functioning receptors in the combinatorial code for the mixture as a whole (Trimmer 2019). Another possibility is that these phenotypes may be multigenic (lines 523-524) and we don’t have the power to resolve multi-gene interactions. To highlight this point,* *the following content has been described in the abstract (****lines 44-48****).*

*“In both populations, consistent with previous studies, we replicated three previously reported associations (β-ionone/OR5A1, androstenone/OR7D4, cis-3-hexen-1-ol/OR2J3 LD-band), but not for odors containing aldehydes, suggesting that olfactory phenotype/genotype studies are robust across populations.”*

*b) Thank you for bringing up this issue. The discovery and validation cohorts had different methods of determining phenotype, including different methods of delivery of the odors, and different concentrations of those odors. It is a strength of the two main novel findings (galaxolide/OR4D6 and 3M2H/OR51B2) that despite these differences in methodology, we found the same variants in both populations (lines 349-351). This suggests that these associations are robust to differences in phenotype measurements.*

*The main differences between the two cohorts arise in the replication of previously found odor associations. For two of these associations (androstenone with OR7D4 RT/WM, and cis-3-hexen-1-ol with OR2J3), the validation study did not find an association, whereas the discovery study did. It is likely that the much smaller validation cohort, was lacking in power to detect these associations, due to a much smaller population studied (n=364 compared to n=1003 for the discovery cohort). This hypothesis is supported by the fact that both of these associations were significant in the meta-analysis of the two cohorts together (lines 281-282, 300-304), since the purpose of a meta-analysis is to increase power by combining data subsets into larger ones. We have added this explanation to our discussion in order to try to clarify reasons for differences in associations between the cohorts (****lines 358-360, 470-472)****.*

2. An issue posed in the prior evaluation is whether the test-retest reliability of odor ratings can be questioned. In my view, the reported reliability of these behavioral measures should be considered surprisingly high in the context of olfactory behavioral measurement. Numbers are also quite similar across samples. In fact, one would only expect higher behavioral test-retest correlations in samples deliberately selected to cover a very wide range of the performance spectrum (e.g. by over-sampling anosmics and hyposmics). High test-retest reliability of about 0.7 makes sense considering the odors are selected based on a highly variable sensitivity in the population – this would imply higher test-retest reliabilities compared to a situation where complex odors are presented at similar intensities. The rank-ordering of odors in each individual might potentially further increase the reliability, as might removing 4 participants who displayed no test-retest correlation. In sum, I find the behavioral assessment to be valid, but the authors may briefly note in the discussion how their methodological decisions led to these results.

*Response: Thank you for the suggestion. We previously mentioned in the methods our decision to rank data and remove the poor test-retest correlation data (lines 602-616). The overall test-retest correlation reported was conducted on the raw, rather than ranked data. In the discovery data, each odor was rated a second time by a unique subset of 100 participants. We are unable to conduct an analysis of the ranked test-retest in this population because no participants had two full second set of data to rank. Therefore, our test-retest calculations were conducted prior to ranking the data. We have clarified this in the methods section (****lines 611-613****). We agree that it is appropriate to address the test-retest values in the context of odors with high phenotypic variability and have added this to our discussion of test-retest values* ***(lines 484-489****).*

3. The odor concentrations were established based on evaluations from 14 expert panelists, but these panelists are not well characterized (page 28). Who were they?

*Response: This panel was a subset of individuals from a trained sensory flavor evaluation panel at Unilever. We have added this detail to the methods section (****lines 624-625).***

4. The authors state that ”the derived SNP alleles cause lower odor intensities”. Such statements should be revised to avoid strong causal inference from what is in principle correlational work, although mechanisms may be assumed. It could also be unpacked for a non-specialist readership - how is it determined that the SNPs are associated with lower, rather than higher, intensities. I assume this is based on the known evolutionary trajectories (the results contrasts odor intensity in variant > reference homozygotes), but this is not clearly explained (e.g. on page 10, in the introduction or abstract) until pages 15 and 37.

*Response: Thank you for this suggestion. While we could not find the exact instance quoted here, we agree that this type of language would be overstating our results. We have edited the manuscript in several places to ensure that we are not implying causality where we have correlative evidence (****lines 56-57, 68-71, 187, 322****).*

*A SNP’s association with higher or lower intensity is determined by the statistical test beta value, which shows the direction of the association (whether it’s a positive or negative correlation). This is highlighted in the genotype/phenotype graphs of Figures 2c, 3c, and 4c. The direction of the correlation then informs the evolutionary analysis, where we examine whether the derived allele is associated with an increase or decrease in intensity. In order to clarify the evolutionary analysis for the readers, we have added a sentence at the beginning of the results section on this analysis (****lines 324-327****), stating “From catalogued data by dbSNP, we extracted information about the evolutionary status of each polymorphism, including the estimated age of when the mutation occurred, and which allele was derived (mutated) versus ancestral.”*

5. The authors report finding novel receptors for musk and a body odor compound. Please clarify briefly (also in abstract) how the association between odor perception and SNPs can only reasonably be explained by resulting variations in olfactory receptors because the targets were found on OR nuclei of the genome - as receptor activity was not directly studied, this would be helpful for a non-specialist to know in the early parts of the manuscript.

*Response: For the body odor compound, we were able to directly study the receptor activity, using a cell-based assay (Figure 4e). A variant in OR51B2 had the highest signal associated with 3M2H (body odor) intensity. We found that when this receptor was placed into a cell, it responded to 3M2H (lines 252-259). Similar experiments conducted with OR4D6 were unsuccessful because Galaxolide is not tolerated by the cell-based assay (lines 226-230, 393-395). In general, if we find a highly associated SNP in the open reading frame of a receptor, it is likely that that receptor, or potentially a nearby receptor in high linkage-disequilibrium, is responsible for the phenotype. This type of study has been conducted previously to examine associations between olfactory receptors and differences in odor perception (references 5-13). We have clarified this section in the introduction to specify cases where odors are associated with an SNP in an OR where there is no direct receptor activity measurement (references 10,11,13;* ***lines 93-94****), but the presence of the SNP in the OR provides genetic evidence that it may be responsible for the phenotype. We also clarify briefly in the abstract that ‘SNPs detected for odor intensity were enriched with amino acid substitutions, implying functional changes of odor receptors.’* ***(lines 53-55)****.*

*The discovery cohort examined a genome-wide search, rather than a targeted OR sequencing strategy in order to see if there are variants outside of olfactory receptors that may be responsible for different odor phenotypes. We discuss that, despite this, our significant associations are all in olfactory receptors, which suggests that the genetic differences in olfactory receptors are most likely to be responsible for odor phenotypes (lines 355-358).*

6. 184-186 Explain the direction of the relationship of perceived intensity and pleasantness – how are these perceptual dimensions linked by genotypc variation (e.g. high intensity = low pleasantness)?

*Response: Thank you for the suggestion. Changing an odor’s intensity can change the odor’s pleasantness, but the direction of the relationship depends both on the odor and on the range of concentrations (1.see citation below). Here, we looked at the direction of the genetic effect for perceived intensity and pleasantness. We find that pleasantness is positively correlated with intensity for Galaxolide and beta-ionone (i.e. more intense = more pleasant), and negatively correlated for 3M2H (TableⅠ).*

*TableⅠ Genetic effects for perceived intensity and pleasantness.*

| *Cohorts* | *SNP* | *Odor* | *A1* | *Beta*  *intensity* | *Beta*  *pleasantness* | *P*  *intensity* | *P*  *pleasantness* |
| --- | --- | --- | --- | --- | --- | --- | --- |
| *discovery* | *rs1453541* | *galaxolide* | *C* | *-1.21* | *-0.37* | *2.07E-25* | *7.24E-03* |
|  | *rs1453542* | *galaxolide* | *C* | *-1.21* | *-0.36* | *2.59E-25* | *9.92E-03* |
|  | *rs10837814* | *3m2h* | *A* | *0.68* | *-0.38* | *6.57E-10* | *3.16E-04* |
|  | *rs6591536* | *beta ionone* | *G* | *1.42* | *0.20* | *1.84E-41* | *6.92E-02* |
| *validation* | *rs1453541* | *galaxolide* | *C* | *-6.64* | *-1.55* | *9.08E-06* | *3.33E-01* |
|  | *rs1453542* | *galaxolide* | *C* | *-8.41* | *-1.67* | *1.02E-05* | *4.20E-01* |
|  | *rs10837814* | *3m2h* | *A* | *9.10* | *-4.35* | *9.60E-08* | *2.34E-03* |
|  | *rs6591536* | *beta ionone* | *G* | *22.58* | *16.59* | *7.82E-39* | *5.53E-19* |

*Our goal in this original sentence (now lines 191-193) was to provide a broad summary of our findings as an introduction to the specific odors we looked at. While we did measure pleasantness phenotypes, the SNPs that were associated with pleasantness were also associated with intensity for the same odors, but the effect was larger for the intensity phenotypes. Therefore, the pleasantness phenotypes did not provide additional information about what receptors were associated with odor phenotypes.*

*In order to address questions about the direction of pleasantness associations, we added a parenthetical (****line 193****) to direct readers to Supplementary data tables 1 and 2, which show the full set of significant hits in the discovery cohort and replication cohort (Supp Data Table 1), and the meta-analysis results (Supp Data Table 2), including the direction of the pleasantness associations (indicated by direction_meta).*

*We thank the reviewer for this comment because it has brought to our attention that we overlooked including raw pleasantness data for some of these associations which are later discussed. We have added a new set of Supplementary Data (Supplementary Data 3;* ***lines 1117-1120****), which shows the meta-analysis data for the set of replication associations. For beta-ionone, androstenone, cis-3-hexen-1-ol, and caproic acid, we looked for associations within +- 200k bp from the replicated association, with alpha=0.05 (for a priori hypothesis of association). This data had not previously been included in the other tables, which include only the GWAS significant associations (p<5e-8).*

*1.Doty, R. An examination of relationships between the pleasantness, intensity and concentration of 10 odorous stimuli. Percept Psychophys* ***17****, 492–496 (1975).*
